# Supplementary material for: 3D Multiple Triangular Prisms for Highly Sensitive Non-Contact Mode Triboelectric Bending Sensors
Source: Nanomaterials (Basel). 2022 Apr 28;12(9):1499. doi: 10.3390/nano12091499 (PMC9102195; doi:10.3390/nano12091499)
Supplement: Supplementary file 1 [file nanomaterials-12-01499-s001.zip › nanomaterials-1657907-supplementary.pdf]

nanomaterials

MDPI

Supplementary Materials

3D Multiple Triangular Prisms for Highly Sensitive Non-Contact Mode Triboelectric Bending Sensors

Gi Hyeon Han [1,2,†], Sun Woo Kim [1,†], Jin Kyeom Kim [1], Seung Hyun Lee [1], Myeong Hoon Jeong [2], Hyun Cheol Song [1,3,4], Kyoung Jin Choi [2] and Jeong Min Baik [1,3,*]

1 School of Advanced Materials Science and Engineering, Sungkyunkwan University (SKKU), Suwon 16419, Korea; ghhan@unist.ac.kr (G.H.H.); kimsunwoo0917@gmail.com (S.W.K.); jingyeom0825@naver.com (J.K.K.); samysh@g.skku.edu (S.H.L.); hcsong@kist.re.kr (H.C.S.)
2 School of Materials Science and Engineering, Ulsan National Institute of Science and Technology (UNIST), Ulsan 44919, Korea; mhjeong0102@unist.ac.kr (M.H.J.); choi@unist.ac.kr (K.J.C.)
3 KIST-SKKU Carbon-Neutral Research Center, Sungkyunkwan University (SKKU), Suwon 16419, Korea
4 Center for Electronic Materials, Korea Institute of Science and Technology (KIST), Seoul 02792, Korea
* Correspondence: jbaik97@skku.edu
† These authors contributed equally to this work.

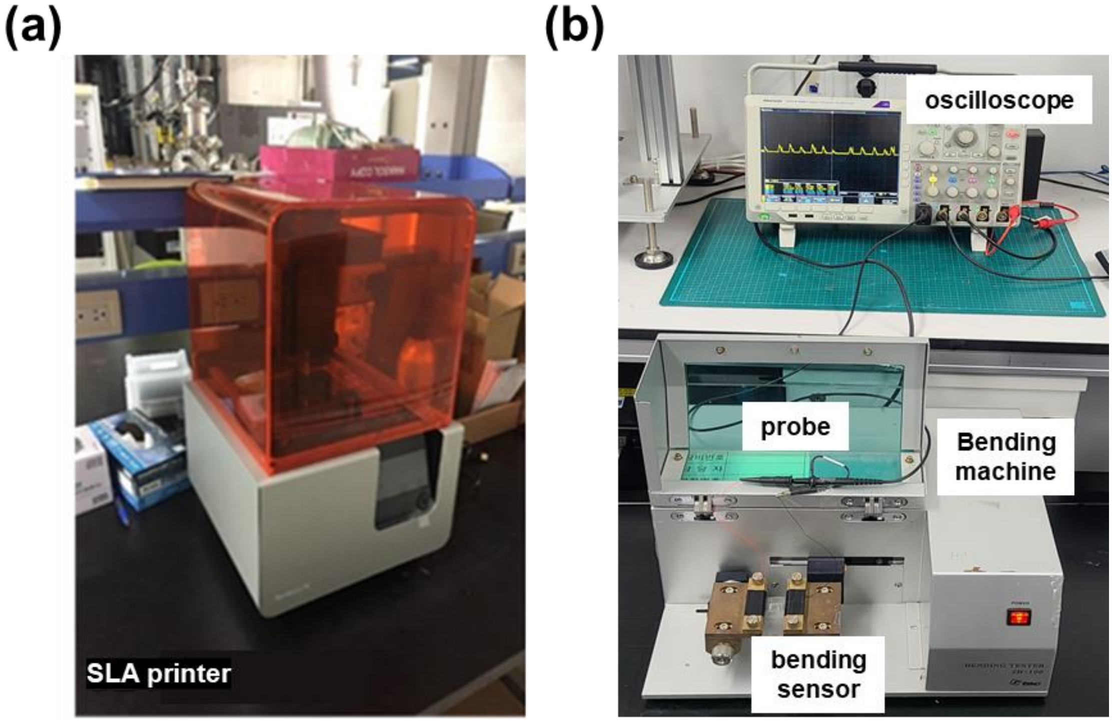

Figure S1. Photographs of the SLA 3D printer (**a**) and electric output signal measurement system (**b**) with bending motion.

Table S1. Parameters of SLA 3D printing.

3D printing parameters	Conditions
Laser spot size (μm)	140
Laser wavelength (nm)	405
Laser power (mW)	250
Bed Temp. (°C)	35
Layer height (μm)	100

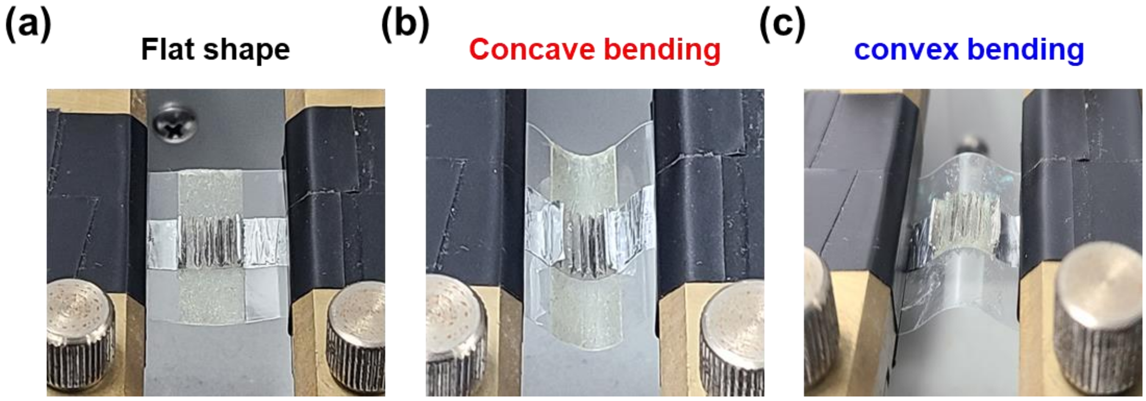

Figure S2. Photographs of sensors with different bending direction. (**a**) flat shape without bending, (**b**) concave bending, (**c**) convex bending.

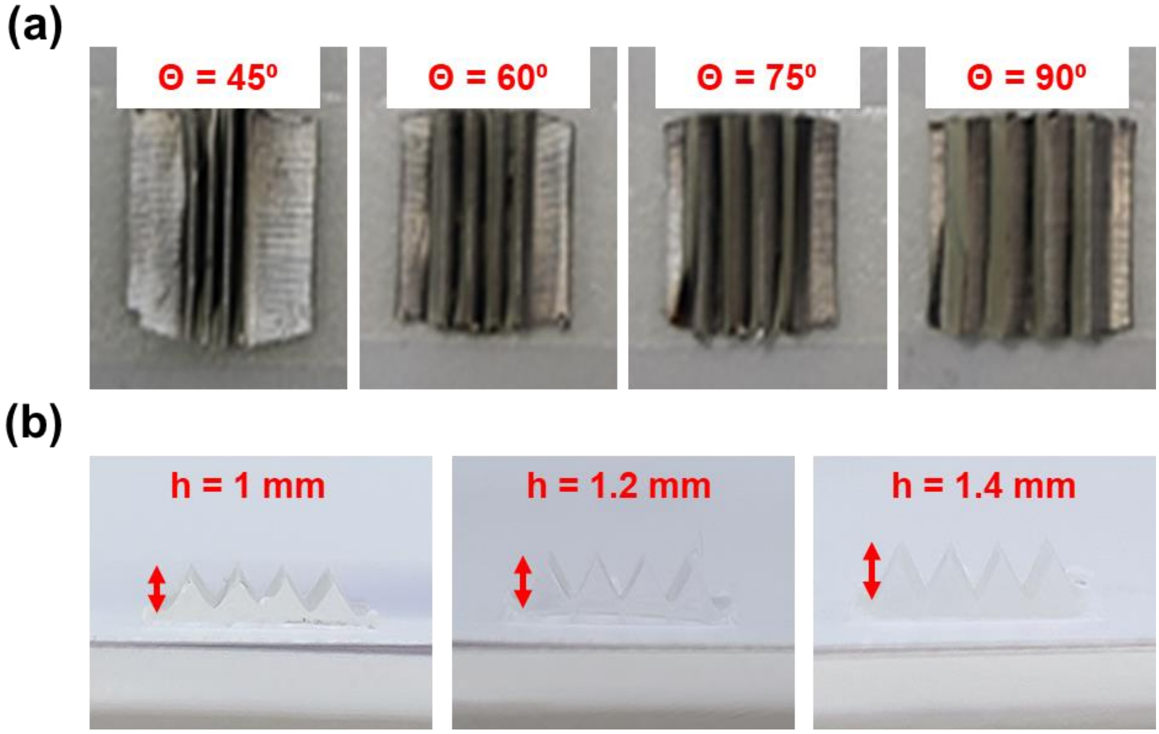

Figure S3. Photos of the sensors with different angles between triangles (**a**) and with different heights (**b**).

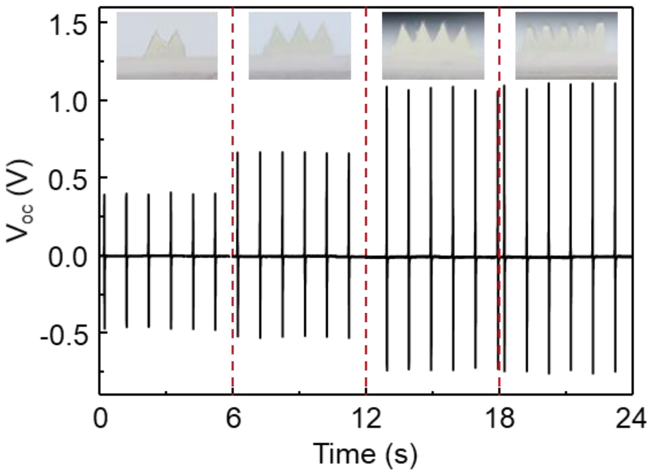

Figure S4. Open circuit voltages of the sensor with the number (2, 3, 4, and 5) of the triangular prisms.

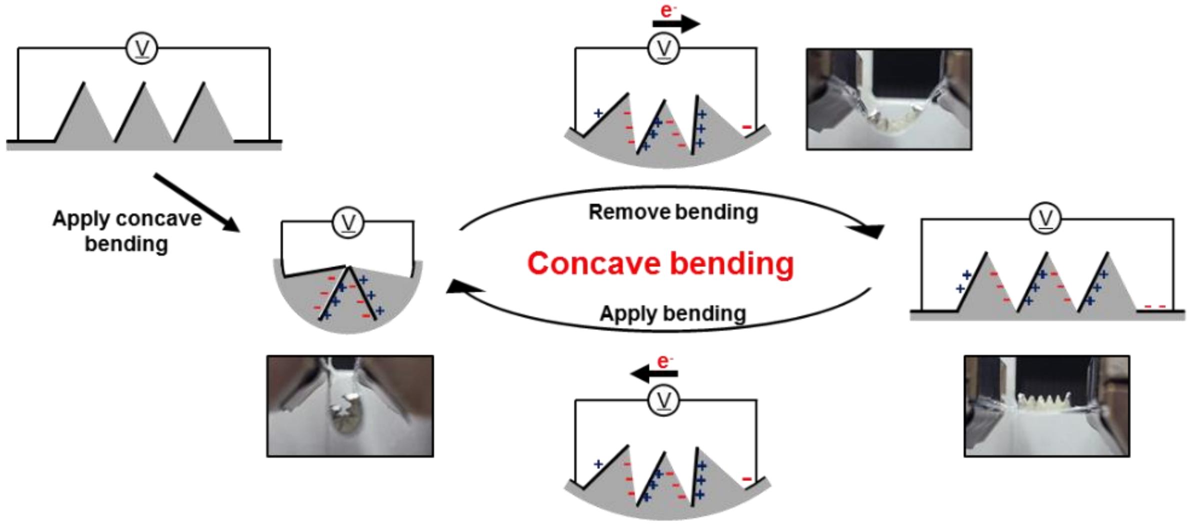

Figure S5. Potential generation mechanism of the sensor by concave bending motion.

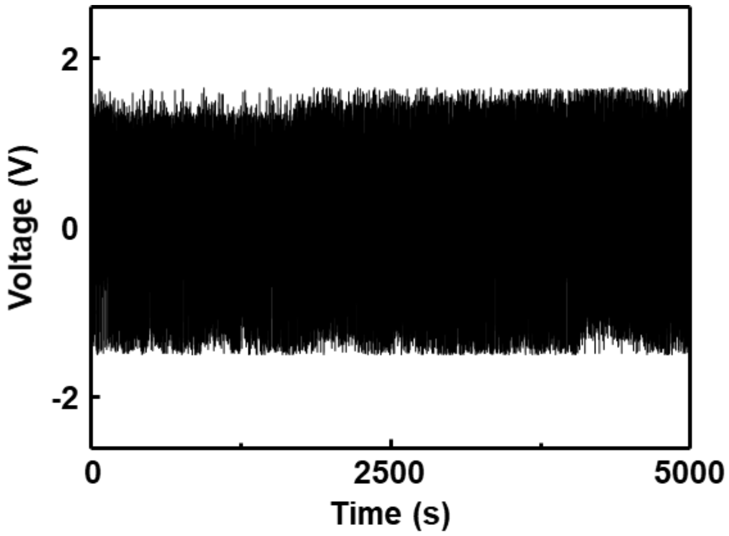

Figure S6. The stability and durability test of bending sensor.

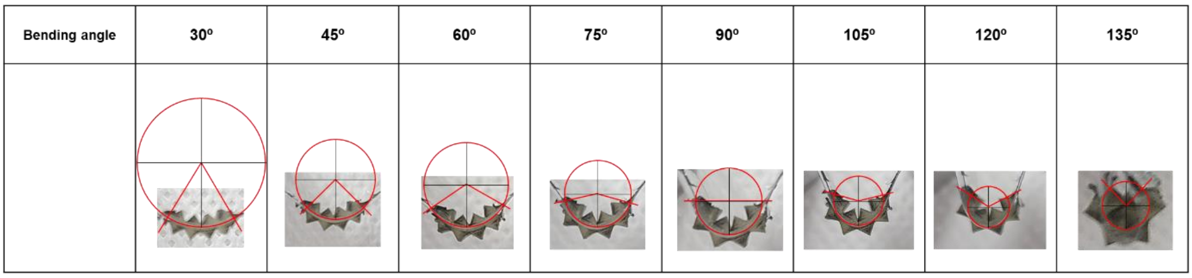

Figure S7. Photograph of sensors with various concave bending from 30° to 135°.

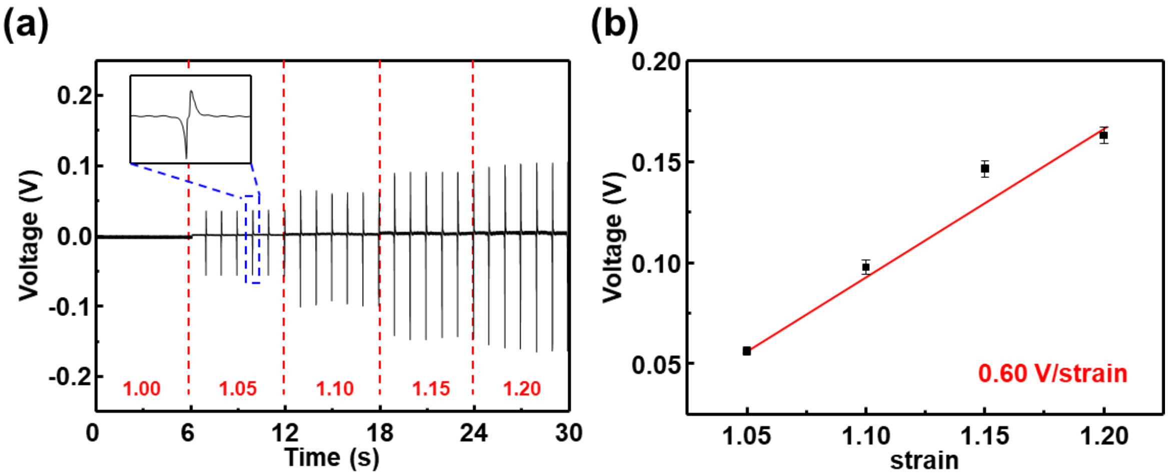

Figure S8. Output voltages of the bending sensor with strain (**a**) and sensitivity (**b**).
